# Graphene-Based Textile Sensors for Intelligent Structural Health Monitoring

**DOI:** 10.3390/polym17111484

**Published:** 2025-05-27

**Authors:** Chuang Zhu, Yajie Zhang, Guanliang He

**Affiliations:** 1 Shanghai Frontiers Science Center of Advanced Textiles, College of Textiles, Donghua University, Shanghai 201620, China; zyj20000620@163.com; 2 Shandong Laboratory of Advanced Materials and Green Manufacturing at Yantai, Yantai 264006, China; 3 School of Materials, University of Manchester, Oxford Road, Manchester M13 9PL, UK

**Keywords:** composites, graphene, sensors, structural health monitoring

## Abstract

This paper presents an approach to address the need for advanced Structural Health Monitoring (SHM) systems in composite materials across various industries. Leveraging the exceptional mechanical and electrical properties of graphene, this study introduces graphene/polydopamine-based sensors for intelligent SHM. Testing these sensors’ efficiency through tensile, bending, impact, and compression tests show that different designs are applicable to various conditions. Notably, these sensors respond well to external impact forces, opening new avenues for impact detection in composite panels, an essential aspect of Structural Health Monitoring. This research contributes to developing resilient and cost-effective SHM systems with potential applications in aerospace, automotive, and civil engineering industries, enhancing safety and reducing maintenance costs.

## 1. Introduction

Composite materials have gained widespread use in various industries, including aerospace, automotive, and bridge engineering, owing to their exceptional mechanical properties and low density [1,2,3]. Examples of this adoption include the Airbus A380 and Boeing B787 aircraft models, where composite materials constitute more than 40% of the entire structure, resulting in a substantial weight reduction of over four tons compared to traditional alloys [4,5,6].

With this rapid adoption comes an urgent need to safeguard long-term performance. Mechanically, wing and fuselage panels routinely experience quasi-static, tensile/compressive stresses of up to 300 MPa as well as in-plane shear stresses of up to 75 MPa, while the fastened joints in these areas can experience bearing stresses of 200–250 MPa [7]. Aircraft structures face various transient hazards, including bird strikes, runway debris impacts, and low-velocity impacts, which can cause significant damage and reduce mechanical performance. Gust loading during flight introduces cyclic bending with peak strain amplitudes of 0.2–0.5% per pressurization cycle [8]. These benchmark figures establish the quantitative loading envelope that any embedded system must withstand to serve as practical and dedicated sensing modes—impact, bending, strain, and pressure. Each sensor can reliably capture the specific mechanical events an aircraft will encounter throughout its service life. Composite structures are susceptible to various forms of stress, including bending, delamination, and stretching, primarily induced by impact forces such as bird strikes, wind, and particle impacts [9,10,11]. These factors present inherent risks to the structural integrity of composite materials, necessitating the development of advanced Structural Health Monitoring (SHM) systems. Early detection of structural deformations and sudden impact forces is vital to prevent catastrophic structural failures and to reduce maintenance costs [12,13].

Traditional SHM techniques for composites often rely on ultrasound-based, non-destructive testing, which involves analyzing changes in ultrasound wave reflection, transmission, and scattering within different composite structures for diagnostic purposes [14,15,16,17]. While ultrasound non-destructive testing is non-invasive, it is characterized by extended inspection times and relatively high operational costs, making it less suitable for routine maintenance. Consequently, integrating embedded sensors represents a promising approach for continuously monitoring composite structure health. However, conventional sensors based on metal and ceramic materials pose challenges when integrated into complex geometric shapes due to their high hardness and potential for inducing failure mechanisms in composites due to inadequate bonding [18,19].

Graphene, a two-dimensional material comprising a single layer of bonded hexagonal carbon atoms, exhibits remarkable mechanical and electrical properties [20,21]. Its two-dimensional nature results in a vast surface area, rendering it highly sensitive to external environmental changes [22]. Graphene sensors show stability, with chemical durability dictating service life, especially in environments alternating between humid air, mild acids, and weak alkaline cleaners [23]. For textiles, Zhou et al. reported that PDA-grafted PET fabrics maintained super-hydrophobicity and tensile integrity after 50 washing cycles in 0.1 M NaOH and 0.1 M H_2_SO_4_ solutions [24]. Nevertheless, pristine graphene faces challenges when applied to substrates in large quantities using traditional methods, as it is chemically stable and inert, making it challenging to adhere to surfaces [25]. Moreover, graphene coatings are prone to abrasion, with minor scratches leading to the detachment of graphene flakes, significantly impairing sensor performance. This study examines polydopamine (PDA)-modified substrates with graphene to address these issues. Polydopamine (PDA) is a substance secreted by mussels during adhesion, displaying excellent adsorption capabilities on a wide range of material surfaces, including polymers, ceramics, and textiles, when coated in an alkaline solution [26,27]. Graphene is incorporated to form crosslinks with dopamine-modified fibers for higher adhesion [28].

While previous GO-based sensors featured suboptimal conductivity due to oxygen atoms, this study introduces a straightforward, cost-effective, and scalable approach for fabricating high-performance, pristine graphene sensors on fiber surfaces modified with a PDA adhesive layer, employing a dip-coating method. Additionally, this research outlines the encapsulation of the graphene sensor’s surface to shield graphene flakes from abrasion, enhancing sensor reliability and stability. The investigation further assesses the sensor’s performance by subjecting the graphene-based composite to impact tests, cyclic tensile, compressive, and bending forces on various flexible and rigid composite materials.

## 2. Materials and Methods

### 2.1. Chemicals and Materials

**Figure 1:**
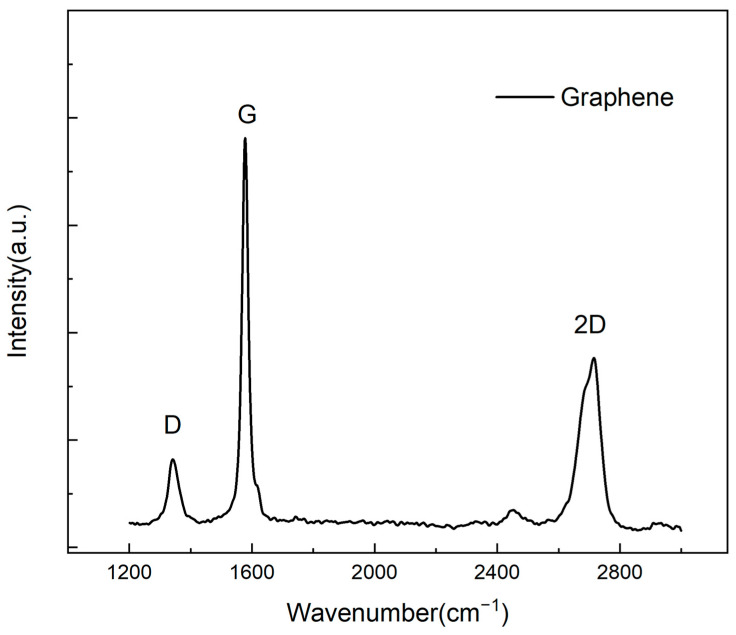
The Raman spectroscopy of graphene.

Graphene ink synthesis was performed at the National Graphene Institute in Manchester, UK, using a micro-fluidization machine (IDEX, Worcester, UK). The Raman spectroscopy of graphene is presented in Figure 1. Raman spectra of the graphene-containing samples were recorded using a 532 nm laser excitation wavelength. The Raman spectra of Graphene show characteristic peaks at ~1352.05 cm^−1^, 1582.53 cm^−1^, and 2727.99 cm^−1^ corresponding to D, G, and 2D bands, which are observed for all samples. The G/2D height ratio is roughly equal to 2:1, which represents the graphene being single layer/few layers. The D band indicates the graphene sample’s disorder, which is due to the edge effect and structural defects introduced to graphene during the micro-fluidization process.

Sensor yarn was created using cotton and E-type glass yarn from the University of Manchester’s Weaving Laboratory (Manchester, UK), with prior preparation involving de-sizing and contaminant removal. The EF80 Flexible Epoxy Resin from Easy Composites, Manchester, UK, produced flexible composites. Furthermore, the 3D-printed composite panel was designed using 1.75 mm diameter PLA 3D Print wire (Yudong, Shenzhen, China).

### 2.2. Sensor Fabrication

**Figure 2:**
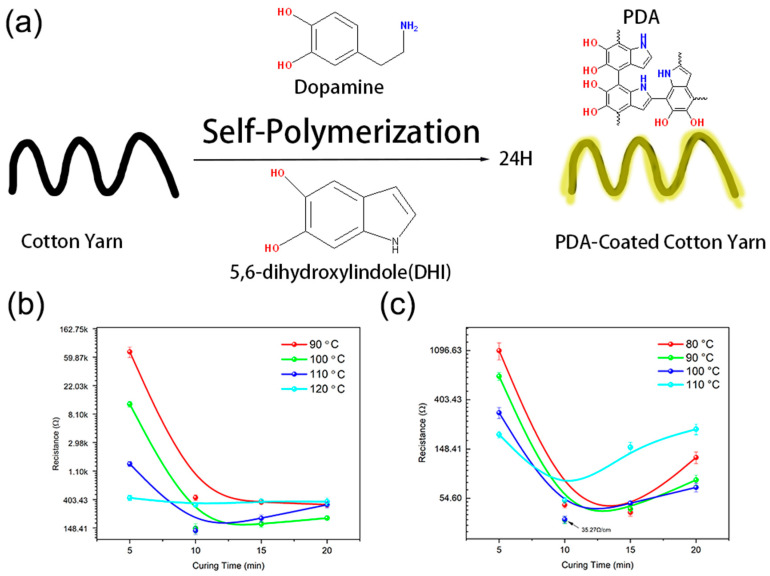
(**a**) Schematic of the processes for preparing dopamine-grafted yarn; (**b**) the resistance of the modified pristine graphene coating on glass yarn as a function of temperature and time; (**c**) the resistance of the modified pristine graphene coating on cotton yarn as a function of temperature and time.

Cotton yarn was used as the substrate and underwent a treatment process involving immersion in a mixture of 5 mg/mL dopamine powder and 1 M/L tris buffer for 24 h. During this period, polydopamine (PDA) was grafted onto the surface of the cotton yarn, as illustrated in Figure 2a. Following this treatment, the sample underwent multiple washes with distilled water (DI) and was subsequently dried using a stream of N_2_ air within a fume cupboard.

The modified cotton yarn was then immersed in a graphene ink bath, followed by a sintering process. Various temperature and curing combinations were examined to optimize the sintering process, with curing times set at 5, 15, 25, and 35 min and temperatures ranging from 90 °C to 120 °C. Figure 2b,c shows the electrical resistance of the glass yarn and the cotton yarn-based, graphene-coated sensors. Initially, the sensor’s resistance significantly decreased with increasing temperature and curing time due to residual solvents’ evaporation. Consequently, the lowest resistances were achieved at 110 °C for 25 min and 110 °C for 15 min for the cotton and glass yarns, respectively. The broader resistance window was observed for the graphene-coated glass yarn than for the cotton yarn. The glass substrate was coated with a carboxymethyl cellulose (CMC)-modified graphene ink. CMC markedly elevates ink viscosity and promotes intimate adhesion to the smooth glass surface, yet its insulating character increases the sheet resistance of the final film, leading to higher absolute and more widely distributed resistance values. By contrast, the cotton yarn was coated with pristine graphene, giving a lower baseline resistance and a narrower range. The longer curing time for the cotton sample was due to the higher graphene content within the yarn, as the yarn is highly hydrophilic and absorbs liquid, while the glass yarn does not absorb as much liquid. It is worth noting that excessively high temperatures could potentially lead to degradation of the cotton fiber structure, crosslinking within macromolecules, and depolymerization. Post-sintering, the cotton yarn samples were sectioned into 10 cm pieces and sealed within PTFE tubes. The morphology of these samples was examined using scanning electron microscopy (SEM).

For impact sensing and piezoresistive sensing within rigid composites, glass yarn was selected as the substrate. The graphene coating for the glass yarn involved the addition of 1 wt% carboxymethyl cellulose (CMC) to pristine graphene, followed by magnetic stirring for one hour.

### 2.3. Preparation of Sensor-Integrated Composite

**Figure 3:**
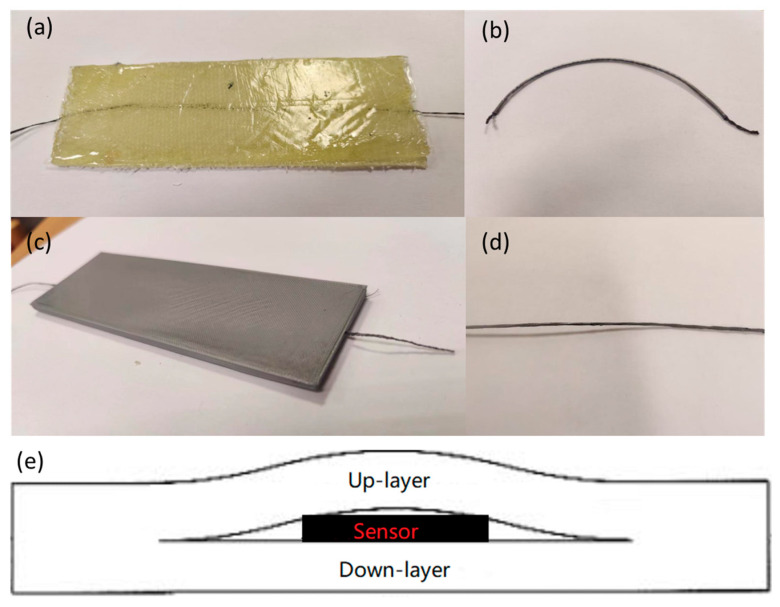
(**a**) Flexible epoxy composites; (**b**) cotton-based, graphene-coated sensor with PTFE tube protector; (**c**) 3D-printed PLA panels; (**d**) glass-based, CMC-modified, graphene-coated sensor; (**e**) the schematic diagram along with the cross-sectional image of the sensors.

Two types of sensor-integrated panels were produced in this research. The cotton-based graphene sensor is encased in a PTFE micro-tube to prevent resin infiltration, thereby retaining high axial strain sensitivity but rendering it insensitive to through-thickness compression or impact. In contrast, the glass-based graphene sensor incorporates a CMC-assisted coating that affords strong adhesion and reliable response under compressive and impact loads, yet it becomes brittle under bending and is therefore unsuitable for flexural tests. Finally, the flexible epoxy laminate dissipates the most impact energy, while the rigid 3D-printed PLA panel is unable to withstand cyclic bending without fracture. The first type involved the creation of a laminated, flexible composite panel through a vacuum bagging process. In this process, the encapsulated graphene-coated sensor was seamlessly integrated into the textile structure of the glass yarn, ensuring a secure fit within the knitted preform. This preform was combined with epoxy resin using a vacuum bagging technique, resulting in the fabrication of flexible composites. These composites were then precisely cut into 12 cm × 5 cm dimensions. The second type of panel was constructed through 3D printing using PLA materials. The internal structure of the panel was printed with a Z-shaped infill with an incorporated groove to accommodate the sensor yarn. During the printing process, a pause was initiated to insert the prepared sensor yarn into the designated groove, and then printing resumed with the yarn now embedded in the design. Four distinct types of samples were successfully manufactured and categorized (Figure 3), as summarized in Table 1.

### 2.4. Characterization

Fourier Transform Infrared Spectroscopy (FTIR) was used to test the samples to confirm that PDA had been successfully coated onto the cotton yarn. Surface morphology was performed using SEM Quanta 650 (FEI, Hillsboro, OR, USA). The tensile test and compression test were carried out only for flexible composite samples integrated with sensor yarns A and C on a Zwick/Roell Z050 (ZwickRoell, Ulm, German). The 3-point bending test (3DP) and cyclic 3-point bending test were carried out using an INSTRON 8802K4935 (Instron, Norwood, MA, USA). The impact test was performed as described in other published works [29].

## 3. Results and Discussion

### 3.1. Surface Morphology

**Figure 4:**
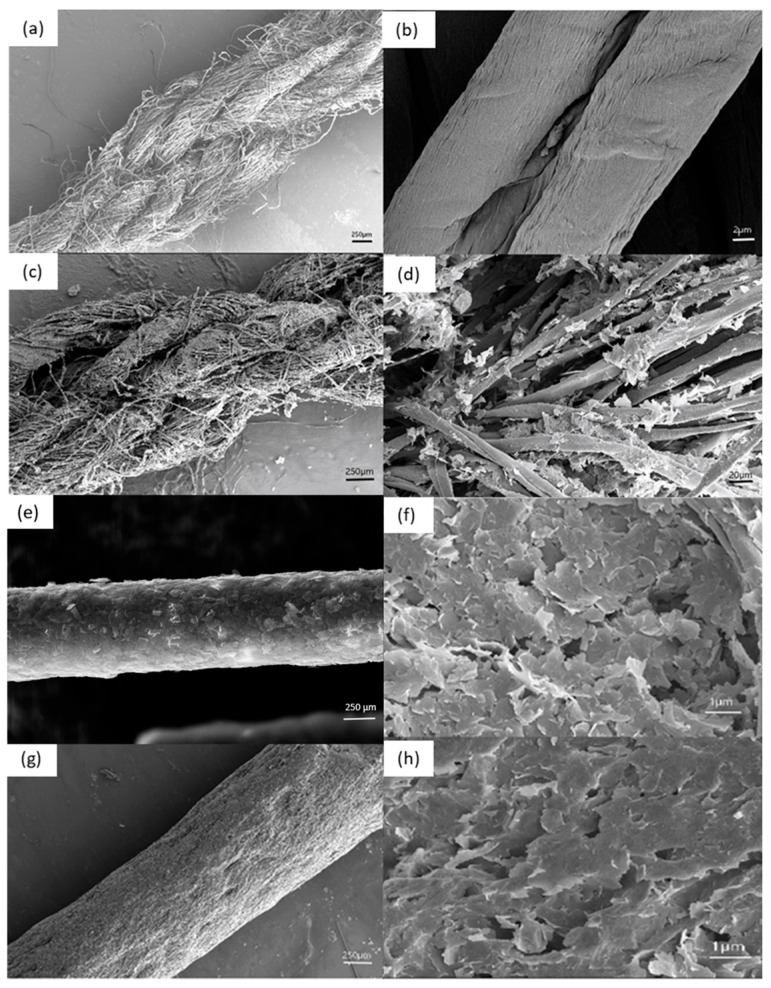
SEM images of surface morphologies for (**a**) pure cotton yarn at 500× magnification, (**b**) pure cotton yarn at 10,000× magnification, (**c**) graphene-coated cotton yarn at 500× magnification, (**d**) graphene-coated cotton yarn at 2000× magnification, (**e**) graphene-coated, dopamine-modified cotton yarn at 5000× magnification; (**f**) graphene-coated, dopamine-modified cotton yarn at 10,000× magnification; (**g**) graphene/CMC-coated glass yarn at 500× magnification; (**h**) graphene/CMC-coated glass yarn at 10,000× magnification.

Figure 4 shows the morphology of untreated cotton, graphene-coated cotton without PDA, and graphene-coated cotton with PDA treatment. The untreated cotton in Figure 4b shows a magnified image of a single fiber, showing its clean surface devoid of impurities, a crucial characteristic for ensuring effective sensor performance. Figure 4c,d shows the cotton yarn coated in graphene without PDA. Here, the flakes use only van der Waals forces to adhere to the cotton yarn, limiting the number of flakes found. Figure 4e,f shows the graphene with PDA, highlighting the significant increase in graphene adhesion. The difference in adhesion strength between Figure 4b,d has implications for subsequent resistance tests, where improved adhesion can enhance sensor performance. Figure 4g,h shows the magnified compression/impact sensors that were made with glass yarn with CMC. This results in a smoother, more uniform graphene coat, which suggests that the added CMC may contribute to a more cohesive and robust graphene structure, which is advantageous for sensor performance in impact and compressive applications.

**Figure 5:**
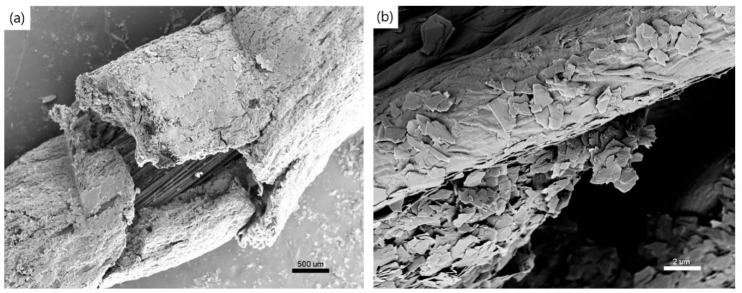
SEM surface morphology of sensor yarns after the tensile test for (**a**) a glass yarn-based sensor at 500× magnification and (**b**) a cotton yarn-based sensor at 5000× magnification.

Figure 5a,b shows the internal fibers of glass- and cotton-based sensor yarns after undergoing tensile testing. In the glass-based sensor yarn, the tensile test strips the graphene coating when the yarn breaks, directly impacting the electrical conductivity. The cotton yarn, in contrast, demonstrates the ability to maintain the integrity of its graphene coating. This observation underscores the potential advantages of cotton-based sensor yarns in applications where strain resistance and electrical conductivity are critical considerations.

Overall, the detailed morphological observations and mechanical test results highlight the significance of material choices and treatment processes in tailoring sensor performance for specific applications. The enhanced adhesion and integrity of graphene coatings on PDA-treated cotton yarns open up new possibilities for improving the reliability and durability of sensors in various contexts, from textile-based strain sensors to impact and compressive sensing applications.

### 3.2. Tensile Test

**Figure 6:**
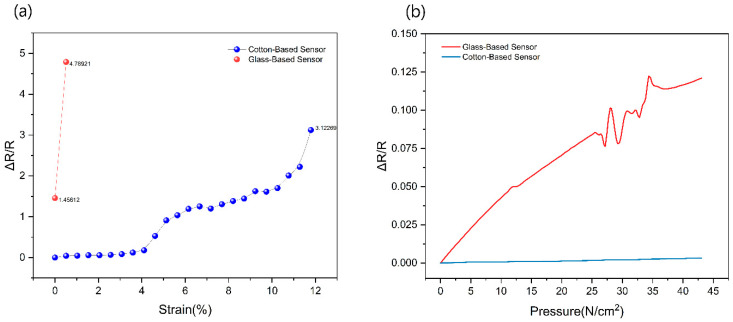
(**a**) Change in resistance vs. strain during the tensile test; (**b**) change in resistance vs. strain during the compression test.

The tensile testing was conducted exclusively on flexible composite samples integrated with sensor yarns A and C. Figure 6a provides a visual representation of the behavior of composites integrated with cotton-based sensors during stretching, highlighting the relationship between electrical resistance and strain. Notably, different strain ranges exhibit distinct slopes in the electrical resistance increase.

For cotton yarn-based sensors, it was observed that the resistance increased gradually when the elongation ranged from 0% to 5%. However, the resistance exhibited a steeper increase when the elongation extended from 5% to 12%.

As seen in the SEM images, many graphene flakes are attached to the outermost layers of the cotton yarn. When the strain is less than 5%, it can be inferred that the outermost layer of graphene flakes moved to fill some gaps between the adjacent flakes. This movement resulted in a small increase in electrical resistance. In the strain range of 5% to 20%, the outermost layer of graphene likely became exhausted, with further straining leading to an expansion of gaps between the graphene flakes, causing the resistance to increase at a more pronounced rate, with the cotton yarn beginning to fracture at 12%.

The magnitude of the change in resistance of the sensor can be regarded as a valuable indicator for assessing and anticipating tensile deformation within the composite. This information can be harnessed to make informed judgments and implement preventive measures in response to tensile stress within the composite material.

### 3.3. Compression Test

When pressure is applied to the sensor, it undergoes deformation, resulting in a flattening effect that increases its surface area. This, in turn, leads to an expansion in the distance between graphene flakes within the sensor, ultimately causing an increase in electrical resistance. Figure 6b shows what occurs as pressure is incrementally applied to the samples. A gradual, linear increase in their electrical resistance was observed. This phenomenon is attributed to the behavior of the sensor when subjected to pressure, which can be quantified through the following equation:

R = Roe + βP(1)

where R represents the resistance of the sensor, Roe is the initial resistance of the sensor, β denotes the sensitivity coefficient of the sensor, and P signifies the applied pressure.

Due to the protective encapsulation of the cotton-based sensor with a PTFE (Polytetrafluoroethylene) tube, sample A’s pressure sensitivity was significantly lower than the glass-based sensor, sample C. The presence of this protective layer results in the absorption of a substantial portion of the applied pressure during the testing process. Consequently, the cotton-based sensor demonstrated limited suitability for a compression sensor requiring sensitivity.

In contrast, the initial resistance of sample C, the glass-based sensor, was approximately 1.2 KΩ. By fitting the experimental data using the formula mentioned above, the sensitivity coefficient β of sample C was determined to be 4 Ω/(g/cm^2^). This sensitivity coefficient quantifies the sensor’s responsiveness to pressure changes. Notably, when compared with the sensitivity of a metallic material-based sensor yarn [30], which exhibited a sensitivity in compression of 2.7 Ω/(g/cm^2^), the glass-based sensor demonstrated higher sensitivity, making it a promising candidate for various pressure-sensing applications where precise and responsive measurements are required.

### 3.4. Bending Test

#### 3.4.1. 3-Point Bending

**Figure 7:**
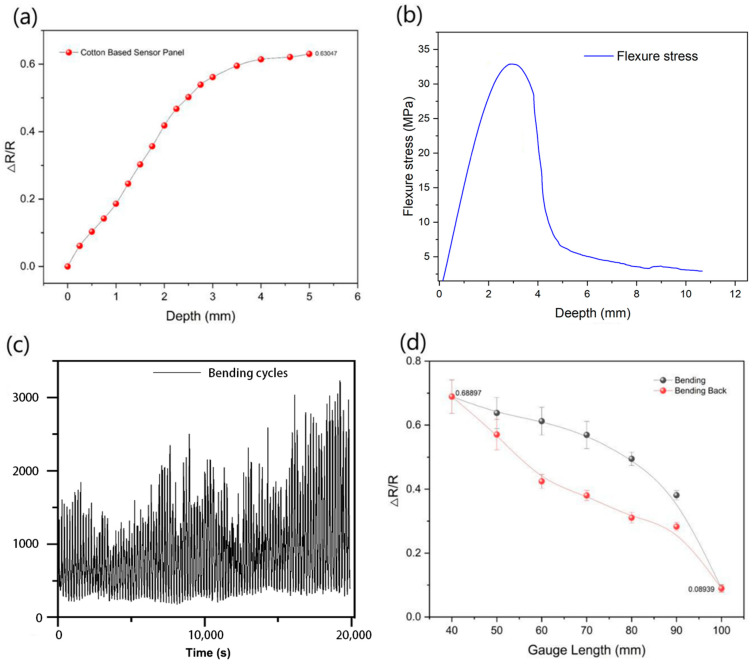
Results from the 3-point bending test, including (**a**) the change in resistance for a single test; (**b**) the stress change for a single test; (**c**) the change in resistance for a cyclic test of a cotton-based sensor; (**d**) the variation in resistance of the sensor in forward and reverse bending.

The panels integrated with sensor sample B underwent a 3-point bending test. Figure 7a illustrates the change in resistance during the test, where the resistance exhibited a linear and uniform increase within a depth range of 0 mm to 3.5 mm. This behavior indicates that sample B was undergoing elastic deformation in this range, with no observable cracking occurring, and the sample retained its ability to return to its original shape after deformation. However, when the bending depth exceeded 4 mm, cracks began to appear in the sample, ultimately leading to a panel fracture.

Figure 7b further illustrates the relationship between bending stress and deformation for sample C. The graph reveals that the bending stress reached its maximum at an approximately 3 mm depth and subsequently exhibited a rapid decline. The sample reached the material’s yield limit in the depth range of 3.5 mm to 4 mm. At this juncture, the applied pressure also decreased, and further compression did not exert a greater force on the sensor due to the inner wall of the PTFE (Polytetrafluoroethylene) tube within the sample. Consequently, the rate of sensor deformation gradually decreased. The reduced slope in the sensor resistance curve elucidates this phenomenon. The 3-point bending test shows the sensor’s utility in monitoring during structural deformations, including elastic and yield deformation and structural fracture events. This capability has significant implications for SHM and damage assessment applications, where early detection of deformation and potential fractures is critical for structural safety and maintenance.

#### 3.4.2. Cyclic Bending Test

To investigate the detection of bending deformation in sensor composite materials over time, a cyclic bending test was conducted on the flexible composite integrated with a sensor, sample A, which utilized a flexible epoxy resin as its matrix, resulting in a high degree of flexibility. After clamping, the sample underwent repetitive bending and unbending movements for 30,000 s.

Figure 7c graphically represents the resistance changes observed in sample A during the cyclic bending test. The resistance increases and then stabilizes after approximately 5000 s. This behavior can be attributed to many closely packed graphene flakes surrounding the cotton yarns at the beginning of the bending test. As the continuous bending and unbending movements took place, the outermost graphene flakes experienced displacement, resulting in an overall increase in resistance. However, in contrast to the outermost flakes, the graphene flakes closer to the surface of the cotton yarn and within the cotton fibers exhibit relatively stable behavior. This stability can be attributed to enhanced adsorption facilitated by the Polydopamine (PDA) treatment. This phenomenon explains the subsequent stabilization of resistance. The long-term experiment demonstrated that the sensor’s performance becomes consistent, affirming its reliability.

Figure 7d quantifies the relationship between the degree of deformation and the rate of change in resistance for sample A. The resistance increased when sample A was subjected to bending and decreased when the sample was straightened. The deformation caused the sensor to become flatter, thereby increasing the distance between graphene flakes and subsequently elevating the electrical resistance. Therefore, by analyzing the resistance value of the sensor, it becomes feasible to roughly determine the degree of bending deformation within the sample. To evaluate the sensor’s energy dissipation characteristics, we adopted the degree of hysteresis (DoH) parameter as defined by Karmakar et al. [31]. We digitized the experimental points displayed in the plot (40–100 mm, 10 mm intervals) and applied the trapezoidal rule. Integration yielded a load = 30.4(ΔR/R)×mm, an unloading = 23.2(ΔR/R)×mm, and a DoH approximately equal to 24%. This result indicates that roughly one quarter of the stored energy is dissipated during unloading, a level generally acceptable for wearable sensor applications.

The cyclic bending test results highlight the sensor’s capability to detect and quantify bending deformations accurately. The initial increase in resistance followed by stabilization depicts the sensor’s reliability and suitability for real-time monitoring of structural deformations and bending events. These findings have significant implications for SHM applications, where the ability to detect and assess bending and deformation events is paramount.

### 3.5. Impact Test

Samples C and D were subject to an impact test where a metal ball is dropped from a height onto the sample. When this happens, it imparts energy to the sample and this impact energy (J) can be quantified by considering the conversion of potential energy to kinetic energy,

fΔt = m√(2gh_0_) (2)

Where fΔt represents the impact energy, m is the mass of the metal ball, g is the acceleration due to gravity (approximated as 10 m/s²), and h_0_ is the height from which the ball was dropped. The three metal balls with different weights had impact energies of 1.5 J, 1.0 J, and 0.5 J, respectively.

**Figure 8:**
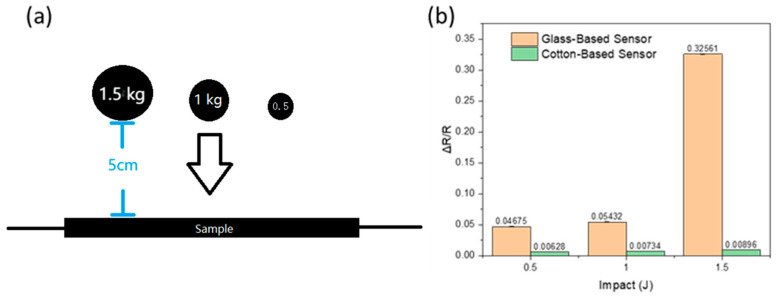
(**a**) Schematic of the ball drop impact test experimental setup; (**b**) the resistance change of sensors during the impact test.

Figure 8a depicts the relative change in resistance for samples C and D under varying impact energies. The resistance changes in the integrated sensors of samples C and D increased in response to the impact energies. Two plausible mechanisms can explain this behavior:

1. Deformation-induced change: When the sample experiences an impact force, it undergoes deformation, resulting in a flattening effect that increases the surface area and expands the distance between graphene flakes. The increased spacing reduces graphene stacking and elevates resistance.
2. Solidification and Cracking: The glass-based sensor in sample D incorporates CMC-mixed graphene, which exhibits a more solid-like behavior. Therefore, under significant impacts, the sensor may experience cracking, leading to a substantial increase in resistance. This behavior results from the impact-induced deformation and the structural response of the sensor material.

Figure 8b shows that while the change in resistance (ΔR) increased from an impact energy of 0.5 J to 1.0 J, the magnitude of this change was not particularly significant. However, when the impact energy was elevated to 1.5 J, ΔR dramatically increased. This rise in resistance suggests the creation of small cracks within the sensor. Furthermore, compared to sample D (comprising a flexible epoxy composite matrix), the resistance change range for sample C was notably smaller. This disparity arises because sample C employed a flexible matrix, which absorbed a significant portion of the impact energy, resulting in a minor change in resistance.

The impact test results indicate that the integrated sensors in samples C and D respond to impact forces by altering their electrical resistance. The observed behaviors can be attributed to the sensor material’s deformation-induced resistance changes and structural responses. Due to its distinctive characteristics and response to impact, Sample D holds promise for applications involving detecting impact forces on composite panels.

## 4. Discussion

Electromechanical responses measured could be applied when benchmarked against the load envelopes outlined above. The cotton-based strain-sensing yarn (Sample A) detected tensile strains from 0% to 12% with two distinct gauge factor regimes (GF_1_ 4.2 for 0–5% strain; GF_2_ 9.8 for 5–12%). Even the lower range gauge factor exceeds the GF_2_ typically required for wing-box strain mapping during gust loading, indicating suitability for distributed in situ strain monitoring in morphing or adaptive wing skins. The glass-based piezoresistive yarn (Sample C) exhibited pressure sensitivity, detecting compressive stresses as low as 5 kPa. When embedded near bolted joints, this resolution is sufficient to track bolt-hole bearing loads that approach 200 MPa in CFRP structures, providing a route to early detection of joint loosening. During impact testing (Sample D), the sensor discriminated impact energies of 0.5, 1.0, and 1.5 J, with a threshold ΔR change of >30% beyond 1 J. This range matches the lower spectrum of soft body impacts (e.g., hail or maintenance tool-drop) on fuselage panels, demonstrating feasibility for sparse impact event detection networks. Combined with the cyclic bending durability proven over 30,000 s, the sensor suite provides comprehensive coverage of dominant mechanical threats faced by composite airframes. Beyond aerospace, the low modulus, encapsulated cotton sensor is attractive for soft robotic skins and wearable health monitoring textiles owing to its survivability under repeated bending to radius 4mm without signal drift. Meanwhile, the rigid 3D-printed PLA panel with glass yarn sensors (Sample B) offers a plug-and-play route for in situ monitoring of additively manufactured drone structures where weight and build process integration are critical. In addition to Structural Health Monitoring, the developed graphene-based sensors show promising potential for wearable applications, given their flexibility, sensitivity, and stability under repeated deformation. Future studies will explore sensor integration into wearable textiles for real-time biomechanical monitoring, such as motion detection and physiological parameter tracking. Further research will focus on optimizing sensor design, assessing biocompatibility and developing ergonomic integration strategies suitable for wearable scenarios.

## 5. Conclusions

Scalable, PDA-assisted dip-coating of pristine graphene has yielded strain-, pressure-, and impact-sensitive yarns that function reliably when embedded in both flexible epoxy and rigid PLA composite panels. Cotton-based yarns encapsulated in PTFE presented gauge factors of 4.2–9.8 within 0–12% tensile strain, whereas CMC-modified graphene on glass yarns exhibited a pressure sensitivity of 4 Ω/(g×cm^−2^)^−1^ and detected impact energies as low as 0.5 J. Across tensile, compression, 3-point bending, cyclic bending, and impact tests, the sensors consistently captured elastic, yield, and fracture events and maintained performance after 30,000 s of repeated loading. These findings demonstrate that a single, cost-effective fabrication route can deliver robust, multimodal sensing elements suitable for Structural Health Monitoring applications in aerospace, automotive, and civil composite structures.

## Figures and Tables

**Table 1:** Types of samples.

	Inner Sensor	Composites Panels	Application
Sample A	Cotton-Based, Graphene-Coated Sensor	Flexible epoxy composites	Tensile/Cyclic Bending Test
Sample B	Cotton-Based, Graphene-Coated Sensor	3D-Printed PLA panels	3-Point Bending Test
Sample C	Glass-Based, Graphene-Coated Sensor	Flexible epoxy composites	Compression Test
Sample D	Glass-Based, Graphene-Coated Sensor	3D-Printed PLA panels	Impact Test

## Data Availability

The original contributions presented in this study are included in the article. Further inquiries can be directed to the corresponding authors.
